# Reasons for Using Cannabis Among Adults in the United States: Associations with Demographics, Health Behaviors, Chronic Conditions, and Legal Status

**DOI:** 10.3390/ijerph23040421

**Published:** 2026-03-27

**Authors:** Ray M. Merrill, Jacob C. Palmer, Henry T. Larson

**Affiliations:** Department of Public Health, College of Life Sciences, Brigham Young University, Provo, UT 84602, USA; palmer63@student.byu.edu (J.C.P.); hlarso24@student.byu.edu (H.T.L.)

**Keywords:** cannabis, chronic disease, legal status, marijuana, medical, recreational

## Abstract

**Highlights:**

**Public health relevance—How does this work relate to a public health issue?**

- This study addresses the growing public health impact of increasing cannabis use in the United States, particularly in the context of expanding legalization and changing social norms.
- It identifies demographic, behavioral, and chronic disease subgroups with differing patterns and motivations for cannabis use, helping to inform targeted prevention and intervention strategies.

**Public health significance—Why is this work of significance to public health?**

- It clarifies how reasons for cannabis use differ across demographic groups, health-risk behaviors, and chronic conditions in a rapidly changing legal environment.
- It provides nationally representative evidence on how legalization status relates to patterns and motivations for cannabis use.

**Public health implications—What are the key implications or messages for practitioners, policy makers and/or researchers in public health?**

- Public health practitioners should incorporate screening and counseling about cannabis use into routine care, particularly for patients with chronic medical conditions.
- Policymakers should consider how legalization and regulation may influence patterns of recreational and medical use across vulnerable populations.

**Abstract:**

**Background:** Several factors influence reasons for cannabis use in the U.S. This study examines reasons for cannabis use (recreational only, medical only, both) and their frequency of use in association with demographic variables, health-risk behaviors, legal status, and chronic disease. **Methods:** We performed a cross-sectional analysis of 466,355 adults (aged ≥18) in the 2018–2021 BRFSS surveys in areas that administered the cannabis module. The primary outcome variables were whether cannabis was used in the past 30 days and, if so, reasons for its use and the number of days of use. Regression techniques were used to assess these outcome measures according to selected variables. **Results:** Approximately 11.5% (SE = 0.1%) used cannabis in the past 30 days. The reasons for use were 36.7% (SE = 0.5%) recreation only, 36.4% (SE = 0.5%) medical and recreation, and 26.9% (SE = 0.4%) medical only. Cannabis use was significantly greater in areas where it was legal for medical and recreational use, but among those who used it, reasons for its use were not significantly associated with legal status. Among those who used cannabis in the past 30 days, using it for recreation only versus medical reasons only was significantly greater in the youngest age group, men, NH Blacks, never married, employed, students, college/technical school graduates, binge drinkers, never smokers, and non-obese and in the years 2020–2021 (vs. 2018–2021). Using it for both medical and recreational reasons versus medical reasons only tended to show similar results. Among those who used cannabis in the past 30 days, the mean number of days of cannabis use was 6.8 (SE = 0.3) days greater for those who used it for medical and recreational reasons vs. recreation only and 5.7 (SE = 0.3) days greater for those who used it for medical reasons only vs. recreation only, after adjusting for several potential confounders. Mean number of days of cannabis use varied significantly across the levels of several variables, including chronic disease status, in the adjusted model. Of those who used cannabis in the past 30 days and had arthritis, asthma, CHD, COPD, depression, diabetes, a heart attack, kidney disease, or cancer, less than half used it for medical purposes only. **Conclusions:** Cannabis use is more common in areas where it is legal for medical and recreational use, but legal status is not significantly associated with reasons for use. Those who use cannabis for medical purposes use it more often than those who use it for recreation only. Reasons for cannabis use vary by the levels of several variables, including chronic disease status. Less than half of those with a chronic disease use it solely for medical purposes.

## 1. Introduction

Over the past decade, cannabis use has increased in the United States and in other parts of the world [1,2]. This has led to important discussions surrounding usage patterns and the motivations behind the consumption of cannabis [3]. Prominent factors contributing to this uptick in cannabis use include changes in cannabis legalization [1], perception of risk [4,5], social norms [6], and availability of high-THC products [4].

Cannabis is a psychoactive drug with a history that dates back 12,000 years [7]. China (2000 BC) and Egypt (1500 BC) have the earliest records of cannabis use for medical purposes [8]. Cannabis became a popular recreational drug in the United States in the early 20th century [7]. In 1970, the U.S. federal government enacted the Controlled Substances Act (CSA), which criminalized the manufacture, distribution, and possession of cannabis [7]. However, over the past few decades, many states, the District of Columbia, and territories have established laws allowing for medical and recreational cannabis use.

In 2024, 24 areas in the U.S. had legalized cannabis for recreational and medical use, and another 14 states had legalized it for medical use only [9]. An increasing number of individuals are consuming high doses of delta-9-Tetrahydrocannabinol (THC), the primary active ingredient of cannabis, for medical and recreational purposes. A recent review has summarized current evidence on risks and medical benefits of cannabis, cannabinoids, and health [10]. The review states that while cannabis is primarily used to experience an acute rewarding effect, regular use of high-THC products can cause addiction and time-limited mental, gastrointestinal, fine motor, and cardiovascular problems. Chronic cannabis use can cause problems of particular concern in adolescents and young adults, which include impaired cognition, disrupted learning, lower educational attainment, anxiety and mood disorders, psychosis/schizophrenia, and suicide. Physical health risks associated with high THC consumption can involve respiratory and cardiovascular issues, prematurity and restricted fetal growth, and cannabinoid hyperemesis syndrome. Herbal cannabis and medicines from extracted or synthesized cannabinoids may produce small to modest benefits for treating chronic pain, muscle spasticity, chemotherapy-induced nausea and vomiting, and refractory epilepsy.

As research continues to educate us on the risks and benefits of cannabis use, a notable gap exists in the literature regarding how reasons for using cannabis (medical, recreational, or both) vary across the levels of several important variables. Reasons behind cannabis use can influence their experience, frequency of use, potential for addiction, and long-term health consequences. Understanding these reasons within subgroups is crucial for clinical applications and improvements in public health policy. The purpose of the current study was to better understand reasons for using cannabis and how these reasons vary according to demographic data, year, health-risk behaviors, BMI weight classifications, cannabis legal status, and chronic disease status. We hypothesized that (1) the reason for cannabis use (recreation only, medical and recreation, medical only) among those who use it in the U.S. differs by legal status, (2) the reasons for using cannabis and the frequency of use vary across the levels of demographic variables, calendar year, health-risk behaviors, legal status, and chronic disease status, and (3) those who use cannabis and have a chronic medical condition use it primarily for medical purposes only.

## 2. Materials and Methods

### 2.1. Data

This study used data from the 2018–2021 Behavioral Risk Factor Surveillance System (BRFSS) surveys. The BRFSS is a nationwide random probability telephone survey that collects individual-level data from U.S. states, territories, and the District of Columbia on health behaviors, chronic health conditions, and the use of preventive services [11].

The BRFSS is conducted by the Centers for Disease Control and Prevention (CDC) to obtain national risk behavior and other health-related data among adults (aged 18 years or older). The BRFSS questionnaire has three parts: (1) core questions involving demographics, health conditions, and behaviors, used by all participating U.S. states, territories, and the District of Columbia; (2) optional modules on specific topics (e.g., cannabis use), which not all areas may choose to consider; and (3) area-added questions for their own specific use [12].

Median response rates for the participating areas were 49.9% in 2018, 49.4% in 2019, 47.9% in 2020, and 44.0% in 2021 [13,14,15,16].

The optional cannabis module was introduced by the BRFSS in 2016 and has since been adopted annually by several U.S. states, territories, and the District of Columbia. In the years 2018 through 2021, BRFSS included a question about why cannabis was used in the past 30 days. The cannabis module and the question about why cannabis was used were adopted by 28 states and territories (Table 1). There were 466,355 individuals who responded to questions about cannabis use in the past 30 days. This number excluded 4258 (0.90%) who answered “don’t know” or “refused” to whether they used cannabis.

This study was exempt from a human subjects research review by the author’s institutional review board because BRFSS data are publicly available and anonymous. Participants are told that they may decline to answer any question. Although BRFSS does not require informed consent for participation, verbal consent is obtained during initial contact. The survey is conducted according to strict CDC protocols, ensuring that all information provided is confidential. A description of the BRFSS survey design, questionnaires, and data collection method is available elsewhere [11,12].

### 2.2. Measures

The cannabis use variable was determined by the question, “During the past 30 days, on how many days did you use marijuana or cannabis?” [17]. If they responded to any use during the past 30 days, they were identified as current cannabis users. The BRFSS treats marijuana and cannabis as synonymous, but for scientific accuracy, “cannabis” will be used throughout the paper. BRFSS asked those who currently used cannabis to specify their primary reason for use: “When you used marijuana or cannabis during the past 30 days, was it usually for (1) medical reasons, (2) non-medical reasons, or (3) for both medical and non-medical reasons.”

Demographic and other variables appear in Table 2. All variables were self-reported. Age group classifications reflected young adults (aged 18–34), middle-aged adults (aged 35–54), and older adults (aged ≥ 55). Sex classifications were men and women. Race/ethnicity classifications were non-Hispanic (NH) White, NH Black, Hispanic, and Other. Marital status classifications were married (combining married [50.8%] with unmarried couples [4.8%]), previously married (divorced [10.8%], widowed [7.2%], separated [2.3%]), never married, and unknown. Employment classifications were employed (employed for wages [47.3%] or self-employed [9.1%]), unemployed (out of work for 1 or more years [2.6%] or out of work for <1 year [31%]), homemaker, student, retired, unemployed, and unknown. Education classifications were less than high school, high school, some college or technical school, college or technical school, and unknown. Heavy drinkers were adult men having more than 14 drinks per week and adult women having more than 7 drinks per week. Binge drinking refers to all types of alcoholic beverages and is defined as men having 5 or more drinks on one occasion and women having 4 or more drinks on one occasion. Smoking classifications were smokes every day, smokes some days, former smoker, never smoker, and unknown. Body mass index (BMI kg/m^2^) weight classifications were underweight (BMI < 18.5), normal weight (18.5 ≤ BMI ≤ 24.9), overweight (25.0 ≤ BMI ≤ 29.9), obese (≥30), and unknown. The legal status of cannabis in each area that participated in the cannabis module was determined by whether cannabis was legal for medical use, recreational use, or neither [18]. The legal status of cannabis in each area was determined by year.

We did not include household income in the study because a high number of participants did not respond to the income question (31.0%) and because of the high collinearity between income and education.

### 2.3. Statistical Analysis

Data were described using frequencies, percentages, means, and standard errors. Estimates were generated using survey strata, primary sampling units, and weights. The Rao–Scott chi-square was used because of the complex survey design to test whether cannabis use and reasons for use varied across the levels of several variables. Binary multiple logistic regression models were used to assess whether reasons for cannabis use (recreation only vs. medical only and medical and recreational vs. medical only) varied across the level of several chronic diseases and other variables. The resulting odds ratio estimates were adjusted for several variables, and Type 3 Effects assessed the statistical significance of each variable, considering the presence of all other variables in the model. The mean number of days of cannabis use in the past 30 days among those who used it varied according to reasons for using cannabis. Whether this result differed across the levels of several chronic diseases and other variables was assessed using regression analysis. Odds ratios were reported with their corresponding 95% confidence intervals (CIs). Statistical significance was present when the CI did not include 1. Statistical significance was based on two-sided hypothesis tests and the α = 0.05 level of significance. To maintain the overall probability of a Type 1 error at 0.05 where multiple comparisons were made, the Bonferroni correction was used, α’ = 0.05/(# of comparisons). Statistical analyses were conducted using Statistical Analysis System (SAS) software, version 9.4 (SAS Institute Inc., Cary, NC, USA, 2016).

## 3. Results

During 2018 through 2021, 40,026 (11.5% [SE = 0.11]) participants used cannabis in the past 30 days. Among this number, 39,563 indicated that they used it for recreation (36.7% [SE = 0.50]), both medical and recreation (36.4% [SE = 0.50]), or just medical reasons (26.9% [SE = 0.45]). There were 463 cannabis users that answered with “Don’t know/Not sure” or “Refused.”

Participants are described according to demographic, health-risk behavior, BMI weight classification, cannabis legal status, and year variables in Table 2. Cannabis use in the past 30 days was significantly greater in younger ages, men, non-Hispanic (NH) Blacks, never married, unemployed or students, with less than a college/technical degree, heavy drinkers, binge drinkers, current smokers, and underweight and in areas where cannabis was legal for recreation and medical use. Recreational-only use of cannabis was significantly greater in the same groups, except unemployed, underweight or obese, and current or former smokers who were less likely to use it for recreation and more likely to use it for medical purposes only. Using cannabis for medical purposes only was significantly more common in older ages, women, NH Whites, previously married, unemployed/homemakers/retired, <high school education, non-heavy drinkers, non-binge drinkers, current or former smokers, and underweight or obese. There was a significant move away from cannabis for medical purposes only over the study period. Legal status was not significantly associated with the reason for cannabis use.

The adjusted odds of recreation only versus medical only and medical and recreational use versus medical only, according to demographic, health-risk behavior, BMI weight classification, cannabis legal status, and year variables, appear in Table 3. Recreational only versus medical only was significantly greater in the youngest and oldest age groups, men, NH Blacks, never married, employed, students, college/technical school graduates, binge drinkers, never smokers, and non-obese and in the years 2020 and 2021. Sex, binge drinking, and then employment status had the strongest associations with the outcome variable. Medical and recreational versus medical use showed similar results, with a few exceptions. Medical and recreational use was no longer significantly associated with college/technical school graduates or with smoking status.

Among those using cannabis in the past 30 days, the mean number of days of cannabis use was significantly greater for recreation and medical (vs. recreation-only) and medical-only (vs. recreation-only) use (Table 4). Mean number of days of use was significantly lower in older ages, women, students, those with a college/technical degree, and binge drinkers and in 2018. Mean use number of days of use was significantly higher in NH Blacks, heavy drinkers, current and former smokers, underweight, and areas where cannabis was legal for recreation and medical use. Interaction terms were assessed between the reason for use and each of the variables in the table. Significant interactions occurred in reason for use by race/ethnicity (F *p* = 0.0116) and reason for use by smoking status (F *p* = 0.0015). NH Blacks had a higher mean score for recreation only (15.0 vs. 11.7 for NH Whites, 10.9 for Hispanics, and 9.7 for Other). Daily smokers had a higher mean score for recreation only (17.3 vs. 13.0 for Occasional, 11.9 for Former, and 9.8 for Never). Daily smokers also had a higher mean score for medical-only use (21.0 vs. 17.1 for Occasional, 18.5 for Former, and 15.8 for Never).

The odds of cannabis use in the past 30 days are shown for selected chronic conditions in Table 5. Cannabis use was significantly more common among individuals with arthritis, asthma, COPD, depression, and cancer (including melanoma), and significantly less common among individuals with diabetes, after adjusting for age, sex, race/ethnicity, marital status, employment, education, legal status, and year.

**Figure 1:**
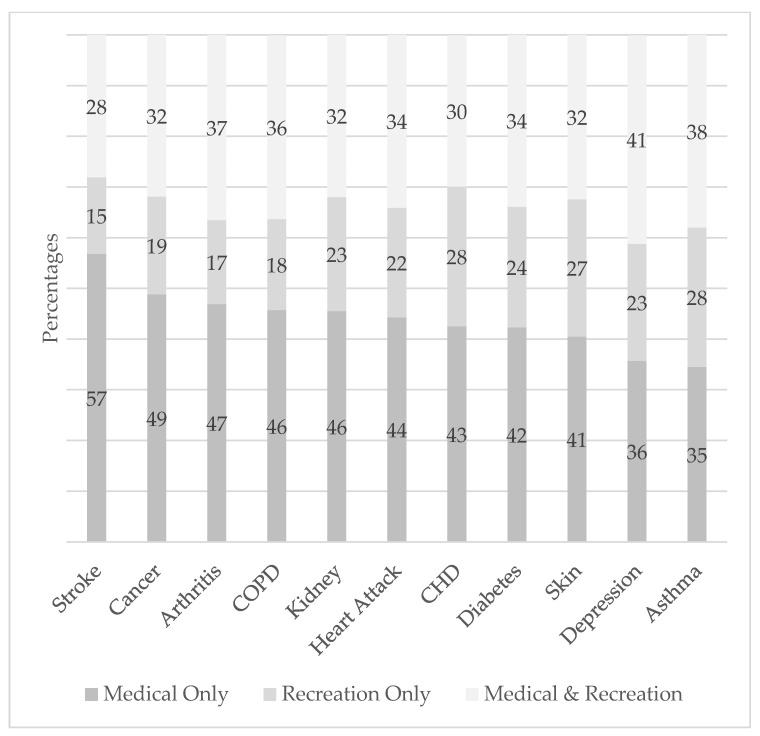
Reasons for cannabis use in the past 30 days by chronic medical condition. Data source: BRFSS. All estimates were weighted based on the complex sampling design.

Among those who used cannabis in the past 30 days, reasons for use were also explored for the selected chronic conditions (Figure 1). The percentage of those who used cannabis for medical-only purposes ranged from 57% for stroke to 35% for asthma. The percentage of those who used cannabis for recreation only was consistently lower than the percentage who used it for both medical and recreational purposes. The adjusted odds of recreation only versus medical only were significantly lower for individuals with arthritis, asthma, COPD, depression, diabetes, a heart attack, kidney disease, stroke, and cancer. The adjusted odds of medical and recreational versus medical only were significantly lower for arthritis, asthma, and stroke.

**Figure 2:**
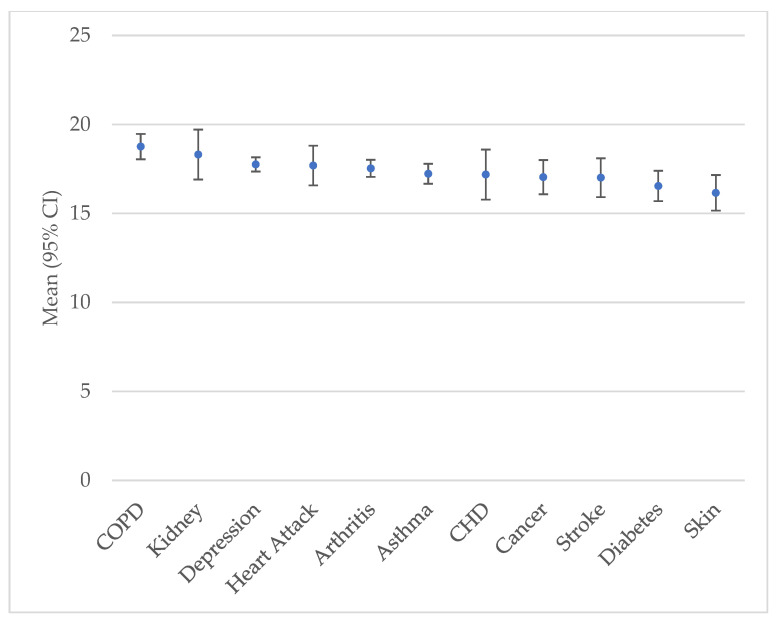
Mean number of days of cannabis use in the past 30 days among those who used it by chronic medical condition. Data source: BRFSS. All estimates were weighted based on the complex sampling design. CI: confidence interval.

Among those who used cannabis in the past 30 days, the mean number of days of use was 17.0 (SE = 0.2) for those with ≥1 chronic disease and 15.1 (SE = 0.2) for those with 0 chronic diseases (*p* < 0.0001). The mean number of days of cannabis use for each chronic disease ranged from 18.8 (SE = 0.4) for COPD to 16.2 (SE = 0.5) for skin cancer (Figure 2).

## 4. Discussion

The study sample represents cannabis use and reasons for consumption in the general U.S. adult population. Consistent with other research, cannabis use decreased with older age [19] and was higher in men [19], NH Blacks [20], never married [21], unemployed [22], less educated [23], heavy drinkers, binge drinkers, and smokers [24,25,26], and underweight [27]. Also consistent with other studies, cannabis use was significantly more common in areas where it was legal for both recreational and medical purposes [28]. Yet legalization has increased young adult use, cannabis-related healthcare visits, impaired driving, negative psychiatric outcomes, and cannabis use disorders [29,30,31,32].

Four specific hypotheses were explored in the current paper, with each discussed here.

**Hypothesis** **1.** *The reason for cannabis use (recreation only, medical and recreation, medical only) among those who use it in the U.S. differs by legal status.*

Despite cannabis use in the past 30 days being significantly greater in areas where it was legal for medical and recreational purposes (vs. medical only or illegal), among those who use cannabis, legal status was not significantly associated with reasons for its use. That is, while legal status influenced level of cannabis use, it did not influence reasons for cannabis use. Around 37% of cannabis users said they used it for recreation only, 37% said they used it for medical and recreational purposes, and 26% said they used it for medical purposes only in the areas represented by each legal classification. Perhaps this is because of the countercultural movement to normalize it, low enforcement and penalties, resilient supply, public health positive messaging, and less perceived risk of it compared with other drugs. In other words, regardless of the law, people are now using cannabis for whatever reason they have [33].

**Hypothesis** **2.** *The reason for using cannabis and the frequency of use vary across the levels of demographic variables, calendar year, health-risk behaviors, legal status, and chronic disease status.*

Reasons for using cannabis significantly varied by each of the variables except legal status. Using cannabis for recreation tended to be more common in those most likely to use cannabis in general (i.e., younger age, men, NH Black, never married, students, heavy drinker, binge drinker), with the exception that unemployed, current and former smokers, and underweight individuals were less likely to use it for recreation and more likely to use it for medical purposes. This is likely because unemployed and underweight individuals and those who smoke cigarettes tend to have poorer health [34,35]. Another exception is that those with a college/technical degree, although significantly less likely to use cannabis, were more likely to use it for recreational purposes. This may be associated with being in better health, so they were less likely to use it for medical purposes, and because they were better able to afford it.

The results suggest that recreational cannabis use is associated with groups who are more socially active, such as alcohol drinkers and students. The higher prevalence of college students reporting using recreational cannabis illustrates how perceptions of recreational cannabis use are becoming more positive. Of note, the higher prevalence of never smokers using recreational cannabis may be because smokers are at greater risk of health problems such as arthritis, where cannabis is increasingly used to lower pain [36,37,38,39,40].

Compared with 2018, use of cannabis for recreation increased significantly in 2020 and 2021, reflecting public perception and policy shifts toward cannabis [4]. Possible reasons for this reflect a combination of factors, including legalization efforts for recreational cannabis and a broadening of the demographic range of users (e.g., older adults and women) [33,41].

Among those who used cannabis in the past 30 days, the mean number of days of cannabis use tended to be higher for those groups where cannabis use was more common (e.g., younger age, men, NH Blacks, students, heavy drinkers, smokers, underweight, and in areas where it was legal for recreation and medical use). Similarly, college/technical school graduates were less likely to use cannabis in the past 30 days, and their mean number of days of use was also lower. Hence, as group acceptance levels decrease, so does the individual level of use. However, students and binge drinkers were more likely to use cannabis in the past month but had a lower number of days of use. Perhaps the demands on students make their cannabis use more sporadic (e.g., weekends), and binge drinking is a substitute for more frequent cannabis use.

The mean number of days of cannabis use in the past 30 days by reason for use only significantly differed by race and smoking status. NH Blacks had a higher mean score for recreation only. Current smokers had a higher mean score for recreation only and medical only. The higher mean score for medical only may be associated with health problems related to smoking.

The mean number of days of cannabis use in the past 30 days also slightly increased (vs. 2018). This corresponds with an increase in use for recreational reasons. Yet, overall, the mean number of days of use was significantly lower for recreation only (11.9) compared to 17.7 for medical only and 18.8 for both recreation and medical purposes. This result is counter to a 2018 study that found that medical-only use of cannabis was the least common reason for use [42]. This shift may be explained by greater acceptance of cannabis for medical purposes and growing use among older adults who are more likely to experience chronic medical conditions. In the outset of this paper, we referred to a study summarizing health risks and benefits associated with cannabis use [10]. A growing knowledge of potential health benefits associated with cannabis use for treating chronic pain, muscle spasticity, chemotherapy-induced nausea and vomiting, and refractory epilepsy may help explain this result.

**Hypothesis** **3.** *Cannabis users with a chronic medical condition use it primarily for medical purposes only.*

Medical use only was the most common reason for cannabis use for each of the conditions except asthma and depression, where medical and recreational purposes were the most common reasons.

Although asthma patients were less likely to use cannabis for recreation only, most cannabis users with asthma used it for both medical and recreation purposes. Perhaps their recreational use has contributed to medical needs. Their asthma may be explained by their cannabis smoking or vaping, which increases the risk of asthma. One study showed a dose–response relationship between cannabis smoking and asthma [43]. A literature review identified cannabis smoking as a precipitating factor for acute asthma [44]. A more recent literature review and meta-analysis also showed that cannabis increased the risk of asthma [45].

The result for depression is likely because of its bidirectional relationship with cannabis. There is some evidence that cannabidiol has antidepressant properties, but randomized clinical trials are needed to confirm this result in humans [46,47,48]. On the other hand, heavy and long-term use of Tetrahydrocannabinol (THC) (the main psychotic component of cannabis) has been shown in longitudinal studies to have a bidirectional association between cannabis use and depression, with cannabis use increasing the risk of depression and vice versa [46]. In a case-control study of lung cancer in adults ≤ 55 in New Zealand, long-term cannabis use was associated with an increased risk of lung cancer [49]. There is limited and conflicting information from studies on the link between cannabis use and various types of cancer [50,51].

Those with arthritis, asthma, COPD, depression, and cancer (excluding skin except melanoma) were more likely to use cannabis, and those with diabetes were less likely to use cannabis, after adjusting for several variables. The result with diabetes may be because cannabis can lower the risk of obesity [27], and because of the positive link between obesity and diabetes [52], those with diabetes are less likely to use cannabis.

Among those who used cannabis in the past 30 days who also had a chronic medical condition, use of cannabis for recreation only ranged from 15% for stroke patients to 28% for CHD patients. Lack of scientific consensus on the effectiveness of cannabis for treating certain chronic conditions may explain why some conditions are associated with greater use for medical purposes and less for recreation only. For example, many stroke patients may perceive cannabis as providing symptom relief, neuroprotective effects, and improved recovery outcomes [53] and so attribute the reason for use more to medical purposes. On the other hand, evidence that cannabis use may help CHD patients is inconclusive, and users are more likely to attribute use to recreation only. Cancer patients were also less likely to indicate using cannabis for recreation only (19%), which may be because some doctors prescribe certain forms of cannabis to help manage common symptoms of cancer and side effects of treatment [54]. However, cannabis may complicate certain treatments such as immunotherapy for cancer [55].

Limitations of this study include not being able to consider causal relationships because of the cross-sectional design, self-reported responses, and chronic medical conditions based on questions regarding lifetime status and not current information about the condition. However, self-reported information about cannabis use, reasons for use, and chronic medical conditions is likely accurate given that BRFSS is an anonymous survey. Yet we should not assume that medical-only use reflects legitimate therapeutic use since BRFSS captures self-reported reasons and not physician recommendation or cardholder status. In addition, cannabidiol and THC can have different effects on the chronic medical conditions considered, but the BRFSS does not provide information on the specific type of cannabis being consumed.

## 5. Conclusions

Reasons for cannabis use vary significantly by demographic and health status, with recreational cannabis use being associated with demographic usage and medical or combined usage being more strongly associated with chronic disease. Reasons for recreational cannabis use vary greatly by geographic legality, age, race, and certain health-risk behaviors. The trends with legalization by state or territory are likely to continue shaping reasons for using cannabis and its prevalence. Medical use among those with chronic health conditions may correlate with increased health burden. Focused clinical screening among those in high-risk groups may be considered to ensure better health outcomes across these demographic groups. Public health communication can be targeted to high-risk groups to educate and inform individuals of health-risk consequences related to cannabis use. Further legislation regarding medical cannabis may be considered for individuals with chronic conditions. As cannabis legalization continues to expand across the U.S., further research should be conducted to determine long-term health impacts.

## Figures and Tables

**Table 1:** Monthly cannabis use by U.S. area and calendar year.

		2018	2019	2020	2021
	No.	%	%	%	%
Alaska	8047	.	.	17.2	20.1
California	18,035	14.9	15.1	.	.
Connecticut	6900	.	.	.	12.1
Delaware	6185	.	.	12.4	11.1
Florida	12,503	10.0	.	.	.
Hawaii	13,974	.	.	11.4	10.8
Idaho	19,273	9.5	9.1	8.8	8.8
Illinois	10,033	.	12.0	10.9	13.8
Indiana	15,562	.	.	9.5	8.8
Kentucky	8175	.	.	10.3	9.4
Maine	21,083	.	.	19.0	21.3
Maryland	57,041	7.9	9.6	9.2	9.0
Minnesota	55,208	9.0	10.3	10.3	9.6
Mississippi	6102	.	.	9.2	.
Montana	10,533	13.7	.	.	14.6
Nevada	2258	.	.	.	18.2
New Hampshire	21,318	9.5	15.1	14.1	13.7
North Dakota	19,710	7.3	7.9	7.0	8.6
Ohio	34,860	9.4	.	12.1	12.3
Rhode Island	9402	.	.	15.8	15.8
South Carolina	18,989	8.6	10.0	9.4	.
Tennessee	12,706	9.5	11.0	9.3	.
Utah	29,831	.	7.6	8.4	8.6
Vermont	5770	.	.	.	21.8
West Virginia	14,784	6.9	10.4	8.3	.
Wyoming	16,230	8.5	9.0	7.3	6.2
Guam	7083	6.3	12.0	8.4	12.7
Puerto Rico	4760	4.9	.	.	.
Total	466,355	11.1	12.6	10.5	11.8

Data source: BRFSS. Percentage estimates were weighted, based on the complex sampling design.

**Table 2:** Cannabis use and reasons for use by selected variables.

	No.	Column % (SE)	Cannabis Use in Past 30 Days % (SE)	Rao–Scott Pr > ChiSq	Recreation Only % (SE)	Recreation and Medical % (SE)	Medical Only % (SE)	Rao–Scott Pr > ChiSq
Age								
18–34	71,608	27.6 (0.2)	20.1 (0.3)	<0.0001	41.6 (0.8)	39.6 (0.8)	18.8 (0.6)	<0.0001
35–54	128,100	32.2 (0.2)	10.8 (0.2)		32.8 (0.9)	35.8 (0.9)	31.4 (0.9)	
≥55	266,647	40.3 (0.2)	6.1 (0.1)		31.0 (0.9)	30.2 (0.9)	38.9 (1.0)	
Sex								
Men	211,995	48.3 (0.2)	14.1 (0.2)	<0.0001	40.2 (0.7)	37.1 (0.6)	22.7 (0.6)	<0.0001
Women	254,360	51.7 (0.2)	9.0 (0.1)		31.5 (0.8)	35.5 (0.8)	33.0 (0.7)	
Race/Ethnicity								
NH White	365,358	64.6 (0.2)	11.1 (0.1)	<0.0001	34.8 (0.6)	36.9 (0.6)	28.4 (0.5)	<0.0001
NH Black	31,971	10.6 (0.1)	15.0 (0.4)		40.4 (1.5)	37.1 (1.5)	22.5 (1.2)	
Hispanic	31,204	16.0 (0.1)	11.3 (0.4)		38.6 (1.6)	36.1 (1.6)	25.3 (1.5)	
Other	37,822	8.9 (0.1)	10.6 (0.4)		41.0 (2.1)	32.8 (1.8)	26.2 (1.7)	
Marital Status								
Married/Cohab	262,974	55.6 (0.2)	8.3 (0.1)	<0.0001	34.2 (0.8)	35.5 (0.8)	30.2 (0.7)	
Previously Married	123,737	20.3 (0.1)	9.8 (0.2)		27.5 (1.0)	34.7 (1.0)	37.9 (1.1)	
Never Married	76,403	23.5 (0.1)	20.5 (0.3)		42.9 (0.8)	38.0 (0.8)	19.1 (0.7)	
Unknown	3241	0.7 (0.0)	7.1 (0.9)		28.0 (5.2)	41.2 (6.3)	30.8 (6.7)	
Employment								
Employed	235,082	56.3 (0.2)	12.6 (0.2)	<0.0001	41.0 (0.7)	37.4 (0.6)	21.6 (0.5)	<0.0001
Unemployed	20,776	5.7 (0.1)	18.4 (0.5)		31.5 (1.5)	41.5 (1.6)	27.1 (1.5)	
Homemaker	20.849	5.5 (0.1)	5.9 (0.3)		20.9 (2.6)	33.5 (2.8)	45.7 (3.1)	
Student	10,624	5.0 (0.1)	17.8 (0.7)		52.2 (2.2)	36.5 (2.1)	11.3 (1.3)	
Retired	146,253	20.3 (0.1)	4.9 (0.2)		29.3 (1.5)	30.1 (1.6)	40.6 (1.6)	
Unable to Work	29,621	5.5 (0.1)	16.1 (0.5)		11.1 (1.0)	32.1 (1.5)	56.8 (1.6)	
Unknown	3150	0.7 (0.0)	7.2 (0.8)		38.1 (5.7)	34.0 (5.7)	28.0 (4.8)	
Education								
<High School	28,271	12.4 (0.1)	10.9 (0.4)	<0.0001	29.3 (1.6)	38.6 (1.8)	32.1 (1.6)	<0.0001
High School	123,283	27.6 (0.1)	12.7 (0.2)		35.2 (0.9)	38.7 (0.9)	26.2 (0.8)	
Some College/Tech	130,406	31.4 (0.2)	13.4 (0.2)		35.7 (0.9)	36.2 (0.9)	28.1 (0.8)	
College/Tech	182,964	28.3 (0.1)	8.4 (0.1)		44.9 (0.9)	32.1 (0.8)	23.0 (0.7)	
Unknown	1431	0.3 (0.0)	6.5 (1.6)		27.5 (10.4)	58.8 (12.1)	13.7 (5.7)	
Heavy Drinker								
No	431,015	92.1 (0.1)	10.3 (0.1)	<0.0001	35.3 (0.6)	36.1 (0.6)	28.6 (0.5)	<0.0001
Yes	26,869	5.9 (0.1)	29.0 (0.6)		42.9 (1.3)	38.3 (1.3)	18.8 (1.0)	
Unknown	8471	2.0 (0.0)	15.6 (0.9)		43.8 (3.3)	36.9 (2.9)	19.4 (2.1)	
Binge Drinker								
No	401,209	83.4 (0.1)	8.5 (0.1)	<0.0001	31.2 (0.6)	35.3 (0.6)	33.5 (0.6)	<0.0001
Yes	56,988	14.7 (0.1)	28.2 (0.4)		46.1 (0.9)	38.2 (0.9)	15.7 (0.6)	
Unknown	8158	1.9 (0.0)	13.7 (0.8)		36.2 (3.5)	39.9 (3.2)	23.9 (2.5)	
Smoking Status								
Daily	45,273	10.1 (0.1)	25.0 (0.4)	<0.0001	29.8 (1.0)	40.1 (1.0)	30.0 (0.9)	<0.0001
Occasional	16,872	4.2 (0.1)	25.3 (0.7)		35.8 (1.6)	37.2 (1.5)	27.0 (1.4)	
Former	128,543	24.3 (0.1)	12.9 (0.2)		29.9 (0.9)	38.1 (1.0)	32.0 (0.9)	
Never	272,909	60.8 (0.2)	7.7 (0.1)		45.1 (0.9)	33.2 (0.8)	21.7 (0.7)	
Unknown	2758	0.5 (0.0)	10.5 (1.4)		33.4 (7.0)	34.9 (6.0)	31.7 (7.4)	
BMI								
Underweight	6841	1.6 (0.0)	18.3 (1.0)	<0.0001	34.6 (3.1)	34.7 (2.8)	30.7 (2.8)	<0.0001
Normal	130,152	28.9 (0.2)	14.7 (0.2)		38.7 (0.8)	36.6 (0.8)	24.7 (0.7)	
Overweight	155,570	32.5 (0.2)	10.9 (0.2)		38.3 (0.9)	36.3 (0.9)	25.4 (0.7)	
Obese	143,262	30.4 (0.1)	10.1 (0.2)		32.6 (1.0)	37.0 (1.0)	30.4 (0.9)	
Unknown	30,530	6.7 (0.1)	4.9 (0.3)		32.4 (2.9)	31.9 (2.8)	35.7 (3.2)	
Legal Status								
Rec and Med	104,893	35.3 (0.1)	14.6 (0.3)	<0.0001	36.9 (0.9)	36.9 (0.9)	26.2 (0.8)	0.5937
Med Only	286,089	48.7 (0.1)	9.9 (0.1)		36.6 (0.6)	35.8 (0.6)	27.6 (0.5)	
Illegal	75,373	16.0 (0.1)	9.5 (0.2)		36.0 (1.1)	36.8 (1.2)	27.2 (1.0)	
Calendar Year								
2018	112,828	31.7 (0.1)	11.1 (0.2)	<0.0001	35.0 (1.0)	36.7 (1.0)	28.3 (0.9)	0.0002
2019	88,223	24.2 (0.1)	12.6 (0.3)		38.3 (1.1)	34.1 (1.0)	27.6 (1.0)	
2020	129,223	23.3 (0.1)	10.5 (0.2)		38.3 (0.9)	35.3 (0.9)	26.5 (0.8)	
2021	136,081	20.7 (0.1)	11.8 (0.2)		35.3 (0.9)	40.1 (0.9)	24.5 (0.8)	

Data source: 2018–2021 BRFSS. SE: standard error. NH: non-Hispanic. All estimates were weighted based on the complex sampling design. Heavy drinkers were adult men having more than 14 drinks per week and adult women having more than 7 drinks per week. Binge drinking considered all types of alcoholic beverages and reflected men having 5 or more drinks on one occasion and women having 4 or more drinks on one occasion. The 12 comparisons were evaluated at the 0.05/12 = 0.004 level.

**Table 3:** Adjusted odds of recreational only versus medical only and medical and recreational versus medical only for selected variables.

	Recreational Only vs. Medical Only		Medical and Recreational vs. Medical Only	
	Adjusted Odds Ratio (95% CI)	Type 3 Effect Pr > F	Adjusted Odds Ratio (95% CI)	Type 3 Effect Pr > F
Age				
18–34	1.00	6.64	1.00	19.22
35–54	0.79 (0.68–0.92)	0.0013	0.69 (0.60–0.79)	<0.0001
≥55	1.02 (0.84–1.23)		0.62 (0.52–0.74)	
Sex				
Men	1.00	91.53	1.00	30.77
Women	0.56 (0.50–0.63)	<0.0001	0.73 (0.65–0.81)	<0.0001
Race/Ethnicity				
NH White	1.00	8.21	1.00	4.28
NH Black	1.59 (1.32–1.92)	<0.0001	1.30 (1.08–1.55)	0.0050
Hispanic	1.03 (0.84–1.26)		0.84 (0.69–1.02)	
Other	1.20 (0.95–1.53)		0.91 (0.75–1.12)	
Marital Status				
Married/Cohab	1.00	11.99	1.00	3.98
Previously Married	0.87 (0.75–1.01)	<0.0001	1.00 (0.87–1.15)	0.0076
Never Married	1.43 (1.23–1.66)		1.25 (1.09–1.43)	
Unknown	0.68 (0.36–1.29)		0.86 (0.44–1.67)	
Employment				
Employed	1.00	52.85	1.00	19.48
Unemployed	0.66 (0.54–0.80)	<0.0001	0.88 (0.74–1.06)	<0.0001
Homemaker	0.42 (0.30–0.59)		0.58 (0.44–0.77)	
Student	1.93 (1.42–2.62)		1.61 (1.19–2.18)	
Retired	0.54 (0.44–0.67)		0.66 (0.54–0.81)	
Unable to Work	0.16 (0.13–0.19)		0.44 (0.37–0.52)	
Unknown	0.59 (0.33–1.05)		0.61 (0.32–1.17)	
Education				
<High School	0.82 (0.65–1.02)	13.08	0.93 (0.76–1.14)	2.75
High School	1.00	<0.0001	1.00	0.0265
Some College/Tech	0.89 (0.76–1.03)		0.89 (0.78–1.02)	
College/Tech	1.42 (1.22–1.64)		1.02 (0.89–1.17)	
Unknown	1.71 (0.67–4.36)		4.50 (1.30–15.61)	
Heavy Drinker				
No	1.00	1.78	1.00	0.07
Yes	1.07 (0.89–1.30)	0.1684	1.03 (0.85–1.24)	0.9366
Unknown	1.51 (0.97–2.33)		1.04 (0.72–1.51)	
Binge Drinker				
No	1.00		1.00	37.85
Yes	2.52 (2.18–2.90)	82.61	1.86 (1.62–2.14)	<0.0001
Unknown	1.20 (0.77–1.88)	<0.0001	1.35 (0.91–2.01)	
Smoking Status				
Daily	0.70 (0.59–0.82)	11.04	1.11 (0.95–1.29)	0.78
Occasional	0.69 (0.56–0.86)	<0.0001	0.97 (0.80–1.19)	0.5410
Former	0.62 (0.53–0.71)		1.00 (0.87–1.15)	
Never	1.00		1.00	
Unknown	0.78 (0.36–1.69)		0.89 (0.49–1.63)	
BMI				
Underweight	0.81 (0.56–1.18)	2.75	0.74 (0.55–0.99)	2.99
Normal	1.00	0.0266	1.00	0.0175
Overweight	1.01 (0.88–1.16)		1.05 (0.92–1.20)	
Obese	0.83 (0.72–0.97)		0.97 (0.85–1.11)	
Unknown	0.72 (0.51–1.02)		0.67 (0.49–0.92)	
Legal Status				
Rec and Med	0.98 (0.83–1.16)	2.47	1.14 (0.98–1.34)	6.06
Med Only	0.88 (0.76–1.01)	0.0848	0.92 (0.81–1.05)	0.0023
Illegal	1.00		1.00	
Calendar Year				
2018	1.00	6.46	1.00	8.65
2019	1.10 (0.93–1.31)	0.0002	1.00 (0.85–1.17)	<0.0001
2020	1.35 (1.16–1.58)		1.14 (0.99–1.32)	
2021	1.31 (1.12–1.53)		1.37 (1.19–1.58)	

Data source: 2018–2021 BRFSS. CI: confidence interval. NH: non-Hispanic. All estimates were weighted based on the complex sampling design. Type 3 analysis of effects considered the statistical significance of each variable considering the presence of all other variables in the table. Odds ratios were derived using multiple logistic regression, with all the variables in the table simultaneously estimated. Heavy drinkers were adult men having more than 14 drinks per week and adult women having more than 7 drinks per week. Binge drinking considered all types of alcoholic beverages and reflects men having 5 or more drinks on one occasion and women having 4 or more drinks on one occasion.

**Table 4:** Adjusted mean number of days of cannabis use in the past 30 days by reasons for using cannabis and selected variables.

	Estimate (SE)	Pr > |t|
Overall	10.1 (0.7)	<0.0001
Reason for Use		
Recreation Only	0.0	
Recreation and Medical	6.9 (0.3)	<0.0001
Medical Only	5.8 (0.3)	<0.0001
Age		
18–34	0.0	
35–54	−1.5 (0.3)	<0.0001
≥55	−3.0 (0.4)	<0.0001
Sex		
Men	0.0	
Women	−1.8 (0.2)	<0.0001
Race/Ethnicity		
NH White	0.0	
NH Black	1.5 (0.4)	0.0002
Hispanic	−0.7 (0.4)	0.0895
Other	−0.5 (0.5)	0.2527
Marital Status		
Married/Cohab	0.0	
Previously Married	−0.4 (0.3)	0.1874
Never Married	−0.4 (0.3)	0.1954
Unknown	2.5 (1.3)	0.0627
Employment		
Employed	0.0	
Unemployed	−0.1 (0.4)	0.7294
Homemaker	−1.0 (0.7)	0.1716
Student	−2.2 (0.5)	<0.0001
Retired	0.9 (0.5)	0.0762
Unable to Work	1.0 (0.5)	0.0391
Unknown	1.1 (1.4)	0.4179
Education		
<High School	0.0	
High School	0.5 (0.5)	0.2738
Some College/Tech	−0.7 (0.5)	0.1207
College/Tech	−2.8 (0.5)	<0.0001
Unknown	−5.3 (3.5)	0.1292
Heavy Drinker		
No	0.0	
Yes	1.8 (0.4)	<0.0001
Unknown	1.6 (1.0)	0.0856
Binge Drinker		
No	0.0	
Yes	−1.1 (0.3)	<0.0001
Unknown	−1.3 (1.0)	0.1783
Smoking Status		
Daily	5.1 (0.3)	<0.0001
Occasional	1.6 (0.4)	0.0003
Former	3.0 (0.3)	<0.0001
Never	0.0	
Unknown	0.1 (1.4)	0.9249
BMI		
Underweight	2.0 (0.7)	0.0027
Normal	0.0	
Overweight	−0.4 (0.3)	0.1516
Obese	−0.6 (0.3)	0.0477
Unknown	0.4 (0.7)	0.5756
Legal Status		
Rec and Med	1.2 (0.4)	0.0010
Med Only	0.6 (0.3)	0.0706
Illegal	0.0	
Calendar Year		
2018	0.0	
2019	1.0 (0.3)	0.0026
2020	0.7 (0.3)	0.0298
2021	1.3 (0.3)	<0.0001

Data source: 2018–2021 BRFSS. SE: standard error. NH: non-Hispanic. All estimates were weighted based on the complex sampling design. Multiple regression estimated the values in the table. Heavy drinkers were adult men having more than 14 drinks per week and adult women having more than 7 drinks per week. Binge drinking considered all types of alcoholic beverages and reflects men having 5 or more drinks on one occasion and women having 4 or more drinks on one occasion.

**Table 5:** Adjusted odds of cannabis use in the past 30 days and, among those who used it, assessment of the reason for use by selected chronic conditions.

					Recreational Only vs. Medical Only		Medical and Recreational vs. Medical Only	
	No.	Column % (SE)	Cannabis Use Odds Ratio (95% CI)	Type 3 Effect Pr > F	Adjusted Odds Ratio (95% CI)	Type 3 Effect Pr > F	Adjusted Odds Ratio (95% CI)	Type 3 Effect Pr > F
Arthritis								
Yes	157,922	26.3 (0.1)	1.52 (1.43–1.61)	98.34	0.26 (0.23–0.30)	172.59	0.70 (0.62–0.80)	15.54
No	305,963	73.1 (0.1)	1.00	<0.0001	1.00	<0.0001	1.00	<0.0001
Unknown	2470	0.5 (0.0)	1.32 (0.99–1.77)		0.45 (0.20–1.04)		0.68 (0.36–1.28)	
Asthma								
Yes	64,651	14.5 (0.1)	1.34 (1.26–1.42)	31.60	0.55 (0.47–0.64)	29.12	0.78 (0.69–0.90)	6.24
No	400,265	85.3 (0.1)	1.00	<0.0001	1.00	<0.0001	1.00	0.0019
Unknown	1439	0.3 (0.0)	0.83 (0.58–1.18)		0.59 (0.28–1.22)		1.04 (0.51–2.16)	
CHD								
Yes	26,710	4.2 (0.1)	1.04 (0.92–1.18)	0.24	0.95 (0.66–1.36)	0.65	0.85 (0.64–1.13)	1.35
No	435,744	95.1 (0.1)	1.00	0.7841	1.00	0.8114	1.00	0.2602
Unknown	3901	0.7 (0.0)	0.98 (0.72–1.34)		1.58 (0.70–3.58)		1.39 (0.80–2.42)	
COPD								
Yes	38,038	6.8 (0.1)	1.58 (1.46–1.72)	61.43	0.50 (0.40–0.63)	19.58	0.84 (0.71–1.00)	2.73
No	426,287	92.7 (0.1)	1.00	<0.0001	1.00	<0.0001	1.00	0.0652
Unknown	2030	0.4 (0.0)	0.87 (0.64–1.18)		0.51 (0.17–1.57)		0.65 (0.34–1.25)	
Depression								
Yes	90,221	19.0 (0.1)	2.36 (2.24–2.48)	360.56	0.43 (0.38–0.48)	82.68	0.88 (0.79–0.98)	2.90
No	374,023	80.5 (0.1)	1.00	<0.0001	1.00	<0.0001	1.00	0.0550
Unknown	2111	0.5 (0.0)	1.37 (1.04–1.80)		0.57 (0.27–1.18)		1.26 (0.64–2.46)	
Diabetes								
Yes	74,839	14.1 (0.1)	0.74 (0.68–0.80)	26.98	0.60 (0.50–0.74)	12.64	0.82 (0.68–0.98)	3.10
No	390,790	85.7 (0.1)	1.00	<0.0001	1.00	<0.0001	1.00	0.0452
Unknown	726	0.2 (0.0)	1.00 (0.58–1.72)		0.69 (0.20–2.40)		2.38 (0.62–9.11)	
Heart Attack								
Yes	26,843	4.4 (0.1)	1.11 (0.99–1.24)	2.27	0.64 (0.48–0.85)	4.74	0.88 (0.69–1.12)	0.74
No	437,283	95.2 (0.1)	1.00	0.1034	1.00	<0.0001	1.00	0.4791
Unknown	2229	0.5 (0.0)	0.83 (0.60–1.14)		0.86 (0.36–2.02)		1.19 (0.68–2.10)	
Kidney								
Yes	18,389	3.2 (0.1)	1.07 (0.93–1.24)	1.50	0.58 (0.39–0.84)	6.52	0.72 (0.52–0.99)	3.88
No	446,348	96.5 (0.1)	1.00	0.2228	1.00	0.0015	1.00	0.0207
Unknown	1618	0.3 (0.0)	0.74 (0.48–1.12)		0.28 (0.09–0.85)		0.45 (0.21–0.99)	
Stroke								
Yes	19,532	3.4 (0.1)	1.19 (1.05–1.36)	4.27	0.35 (0.25–0.48)	20.82	0.54 (0.42–0.68)	14.23
No	445,611	96.3 (0.1)	1.00	0.0140	1.00	<0.0001	1.00	<0.0001
Unknown	1212	0.2 (0.0)	1.30 (0.83–2.04)		0.52 (0.14–1.94)		0.34 (0.11–1.06)	
Cancer								
Yes	46,678	7.2 (0.1)	1.20 (1.10–1.32)	10.42	0.49 (0.39–0.63)	27.44	0.72 (0.58–0.89)	4.64
No	418,557	92.6 (0.1)	1.00	<0.0001	1.00	<0.0001	1.00	0.0097
Unknown	1120	0.2 (0.0)	1.69 (1.11–2.57)		0.11 (0.04–0.28)		1.19 (0.47–2.99)	
Skin								
Yes	46,260	6.8 (0.1)	1.12 (1.01–1.24)	2.80	0.78 (0.61–0.99)	2.04	0.92 (0.72–1.18)	3.72
No	418,804	93.0 (0.1)	1.00	0.0611	1.00	0.1300	1.00	0.0243
Unknown	1291	0.2 (0.0)	1.30 (0.79–2.13)		0.94 (0.36–2.46)		0.32 (0.14–0.74)	
Any Chronic Medical Condition								
Yes	297,531	56.5 (0.2)	1.74 (1.66–1.83)	241.35	0.38 (0.34–0.44)	145.50	0.75 (0.66–0.85)	12.66
No	168,737	43.5 (0.2)	1.00	<0.0001	1.00	<0.0001	1.00	<0.0001
Unknown	87	0.01 (0.0)	0.43 (0.09–2.10)		--		--	

Data source: 2018–2021 BRFSS. CI: confidence interval. CHD: Coronary Heart Disease. COPD: Chronic Obstructive Pulmonary Disease. All estimates were weighted based on the complex sampling design. Separate multiple logistic regression models were derived for each chronic condition, with the odds ratios adjusted for age, sex, race/ethnicity, marital status, employment, education, legal status, and year. Heavy drinkers were adult men having more than 14 drinks per week and adult women having more than 7 drinks per week. Binge drinking considered all types of alcoholic beverages and reflects men having 5 or more drinks on one occasion and women having 4 or more drinks on one occasion. The 11 comparisons were evaluated at the 0.05/11 = 0.005 level.

## Data Availability

Data used in this study is publicly available through the CDC’s BRFSS program: https://www.cdc.gov/brfss/index.html, accessed on 25 February 2026.

## Abbreviations

The following abbreviations are used in this manuscript:

BRFSS	Behavioral Risk Factor Surveillance System
CDC	Centers for Disease Control and Prevention
CHD	Coronary Heart Disease
CI	Confidence Interval
COPD	Chronic Obstructive Pulmonary Disease
NH	Non-Hispanic
SE	Standard Error
SAS	Statistical Analysis System
